# ﻿Remarks on the genus *Phymatodiscus* Berlese, 1917, with the description of Phymatodiscidae fam. nov. and *Bardizoneotvosi* gen. nov., sp. nov. from Indonesia (Acari, Mesostigmata)

**DOI:** 10.3897/zookeys.1182.109744

**Published:** 2023-10-17

**Authors:** Jenő Kontschán, Sergey G. Ermilov

**Affiliations:** 1 HUN-REN Centre for Agricultural Researches, Plant Protection Institute, H-1025, Budapest, PO Box. 102, Hungary HUN-REN Centre for Agricultural Researches, Plant Protection Institute Budapest Hungary; 2 Department of Plant Sciences, Albert Kázmér Faculty of Mosonmagyaróvár, Széchenyi István University, Vár square 2., H-9200 Mosonmagyaróvár, Hungary Széchenyi István University Mosonmagyaróvár Hungary; 3 Institute of Environmental and Agricultural Biology (X-BIO), Tyumen State University, Lenina str. 25, 625000 Tyumen, Russia Tyumen State University Tyumen Russia

**Keywords:** Soil mites, South-East Asia, taxonomy

## Abstract

Phymatodiscidae**fam. nov.** is diagnosed, with *Phymatodiscus* as the type genus. A new genus, *Bardizon***gen. nov.**, with *Bardizoneotvosi***sp. nov.** (from Indonesia) as the type species, is erected to accommodate the *Phymatodiscus* species with an eye-like dorsal depression. Six *Phymatodiscus* species are reclassified as *Bardizon*: *B.aokii* (Hiramatsu, 1985), **comb. nov.**, *B.haradai* (Hiramatsu, 1985), **comb. nov.**, *B.oculatus* (Hirschmann, 1977), **comb. nov.**, *B.kuni* (Kontschán & Starý, 2011), **comb. nov.**, *B.insolitus* (Kontschán & Ripka, 2016), **comb. nov.**, and *B.malayicus* (Kontschán & Starý, 2012), **comb. nov.** The new species differs from the previously described congeners in the sculptural pattern, the shapes of the dorsal and ventral setae, and the sculptural pattern of the sternal shield of the male and the genital shield of the female. A list of all known phymatodiscid species is presented. *Phymatodiscustitanicus* (Berlese, 1905) is moved to the genus *Bostocktrachys*: *B.titanicus* (Berlese, 1905), **comb. nov.** (family Trachyuropodidae).

## ﻿Introduction

The genus *Phymatodiscus* was erected by Berlese (1917) for *Discopomamiranda* Berlese, 1905. Decades later, Hirschmann (1977) revised this genus and transferred two previously described species, *Discopomaconifera* Canestrini, 1897 and *Trachyuropodatitanica* Berlese, 1905, to this taxon. In parallel to the new combinations, Hirschmann (1977) also described four new species from New Guinea. A couple of years later Hiramatsu (1979) described a new species from Japan and two new species from the island of Borneo, Indonesia (Hiramatsu 1985). After a long hiatus, Kontschán and Starý (2011, 2012) described a new species from Vietnam and another new species from Malaysia, and Kontschán and Ripka (2016) discovered and described a new species from Singapore.

In recent years numerous contributions have added more than 30 new species to the Uropodina mite fauna of Southeast Asia (e.g. Kontschán and Starý 2011, 2012; Kontschán and Kiss 2015; Kontschán and Ripka 2016; Kontschán 2018, 2021; Kontschán and Ermilov 2023a, 2023b, 2023c), but knowledge of this group is still far from complete. The present paper contributes towards our understanding of a poorly investigated group of Uropodina mites from Southeast Asia and is based on the collections of the Natural History Museum in Geneva, Switzerland.

## ﻿Materials and methods

The specimens of the new species were cleared in lactic acid for a week and afterwards, investigated on half-covered deep slides with a Leica 1000 microscope. Drawings were made with the aid of a drawing tube on a Leica 1000 microscope. Photographs were taken with Keyence 5000 digital microscope. All specimens are stored in 75% ethanol and deposited in the Natural History Museum in Geneva. All measurements and the scale bars of the figures are given in micrometres (μm).

### ﻿Abbreviations

Setae and pores: ***h*** = hypostomal setae, ***st*** = sternal setae, ***ad*** = adanal setae, ***ps*** = post-anal seta, ***p*** = pores, ***lf*** = lyriform fissures.

## ﻿Systematics

### ﻿ Phymatodiscidae fam. nov.

Taxon classificationAnimaliaMesostigmataTrachyuropodidae

DD822C89-BBFB-55DD-BB13-4D9A31228F73

https://zoobank.org/28862C32-19BC-4A8F-A157-10F1070E16AA

Phymatodiscidae Hirschmann, 1979: 69 (*nomen nudum*).
Phymatodiscidae —Halliday 2016: 355.

#### Type genus.

*Phymatodiscus* Berlese, 1917.

#### Diagnosis.

Idiosoma oval, dorsal shield fused with marginal shield in anterior area. Central area of dorsal shield elevated from neighbouring regions and subdivided with a transversal furrow in longer apical and shorter caudal parts. Transversal furrow forms a pair of eye-like depressions in some species. Genital shield of female scutiform; genital shield of male rounded and situated between coxae IV. Prestigmatid part of peritreme hooked. Corniculi horn-like; internal malae longer than corniculi and densely pilose. Gnathosomal setae in one longitudinal row; *h1* near anterior margin of gnathosoma; setae *h2*, *h3*, and *h4* far from setae *h1* and near each other. Setae *h1* smooth and needle-like; *h2* short and robust; *h3* long and smooth or serrate; *h4* divided into two or three short, serrate branches. Chelicerae with 1–3 teeth on both digits; internal sclerotized pore associated with levantor tendon present. Setae *v1* on palp trochanter long, pilose.

#### Distribution.

All known phymatodiscid species occur in New Guinea, Indonesia, Malaysia, Vietnam, and Singapore.

#### Remarks.

Hirschmann (1979) first introduced a family name, Phymatodiscidae, but it was simply listed and without formal description, diagnosis, or designation of a type genus. Following Halliday’s (2016) suggestion about Hirschmann’s family name, a *nomen nudum*, we maintain the original name but formally establish it here as a new family. Phymatodiscidae currently includes two genera.

### ﻿Genus Phymatodiscus

Taxon classificationAnimaliaMesostigmataTrachyuropodidae

Berlese, 1917

F0C246F2-3088-5C79-B52D-02AB9C36D781

Discopoma (Phymatodiscus) Berlese, 1917: 12.

#### Type species.

*Discopomamiranda* Berlese, 1905: 159, by original designation.

#### Diagnosis.

Phymatodiscid species lacking a pair of eye-like dorsal depressions. Margins of idiosoma with or without many prolongations.

##### ﻿List of the known species

**Remarks.** One species, *Phymatodiscustitanicus* (Berlese, 1905), is transferred here from the family Phymatodiscidae to the family Trachyuropodidae Berlese, 1917. According to the dorsal characteristics (only these were illustrated by Berlese 1905: fig. 13), our opinion is that this species belongs to the genus *Bostocktrachys*, as *B.titanicus* (Berlese, 1905) comb. nov., given that Berlese’s species has strongly sclerotized idiosoma and a deep transversal furrow on the dorsal shield (Kontschán and Ermilov 2023c).

***Phymatodiscusconiferus* (Canestrini, 1897)**

*Discopomaconifera* Canestrini, 1897: 461, 470.

*Phymatodiscusconiferus*—Hirschmann 1977: 60–61.

**Occurrence and biology.** This species has been found in New Guinea, but its habitat is unknown (Canestrini 1897).

***Phymatodiscusignesemovens* Hirschmann, 1977**

*Phymatodiscusignesemovens* Hirschmann, 1977: 64.

**Occurrence and biology.** This species has been found in New Guinea, but its habitat is unknown (Hirschmann 1977).

***Phymatodiscusiriomotensis* Hiramatsu, 1979**

*Phymatodiscusiriomotensis* Hiramatsu, 1979: 108–109.

**Occurrence and biology.** This species was described from leaf litter in Japan (Hiramatsu 1985).

***Phymatodiscusmirabilis* Hirschmann, 1977**

*Phymatodiscusmirabilis* Hirschmann, 1977: 64–65.

**Occurrence and biology.** This species has been found in New Guinea, but its habitat is unknown (Hirschmann 1977).

***Phymatodiscusmirandus* (Berlese, 1905)**

*Discopomamiranda* Berlese, 1905: 159.

Discopoma (Phymatodiscus) miranda—Berlese 1917: 12.

*Trachyuropodamiranda*—Hirschmann and Zirngiebl-Nicol 1967: 21.

*Phymatodiscusmirandus*—Hirschmann 1977: 60–61.

**Occurrence and biology.** This species has been found in Java, Indonesia, but its habitat is unknown (Berlese 1905).

***Phymatodiscuspolyglottis* Hirschmann, 1977**

*Phymatodiscuspolyglottis* Hirschmann, 1977: 63–64.

**Occurrence and biology.** This species has been found in New Guinea, but its habitat is unknown (Hirschmann 1977).

### ﻿ Bardizon gen. nov.

Taxon classificationAnimaliaMesostigmataTrachyuropodidae

1B923450-46E1-5ED8-98DB-C552D71FDEBF

https://zoobank.org/D896ACBC-5F95-4111-8362-F9A395FEC90D

#### Diagnosis.

Phymatodiscid species with one pair of eye-like dorsal depressions.

#### Type species.

*Bardizoneotvosi* sp. nov.

#### Etymology.

The name was suggested by the older son of the first author and derives from small chocolates, which are similar in shape to the idiosoma of these mites.

#### Gender.

Male.

##### ﻿List of the known species

***Bardizonaokii* (Hiramatsu, 1985) comb. nov.**

*Phymatodiscusaokii* Hiramatsu, 1985: 270–273.

**Occurrence and biology.** This species has been described from soil from Borneo (Indonesia) (Hiramatsu 1985).

***Bardizonharadai* (Hiramatsu, 1985) comb. nov.**

*Phymatodiscusharadai* Hiramatsu, 1985: 273–275.

**Occurrence and biology.** This species has been described from soil from Borneo (Indonesia) (Hiramatsu 1985).

***Bardizonoculatus* (Hirschmann, 1977) comb. nov.**

*Phymatodiscusoculatus* Hirschmann, 1977: 62–63.

**Occurrence and biology.** This species has been found in New Guinea, where its habitat is unknown (Hirschmann 1977).

***Bardizonkuni* (Kontschán & Starý, 2011) comb. nov.**

*Phymatodiscuskuni* Kontschán & Starý, 2011: 15–16.

**Occurrence and biology.** This species was collected in Vietnam, in a tropical rain forest (Kontschán and Starý 2011).

***Bardizoninsolitus* (Kontschán & Ripka, 2016) comb. nov.**

*Phymatodiscusinsolitus* Kontschán & Ripka, 2016: 292–296.

**Occurrence and biology.** This species was found in Singapore, where it was collected from soil (Kontschán and Ripka 2016).

***Bardizonmalayicus* (Kontschán & Starý, 2012) comb. nov.**

*Phymatodiscusmalayicus* Kontschán & Starý, 2012: 184–188.

**Occurrence and biology.** This species was collected in Malaysia from leaf litter (Kontschán and Starý 2012).

### ﻿ Bardizon eotvosi sp. nov.

Taxon classificationAnimaliaMesostigmataTrachyuropodidae

B26B4A25-F7D8-5E92-A414-74A80985BAE9

https://zoobank.org/7146CC65-14C0-4BCC-91E0-3C6BDC5B3268

Figs 1 , 2 , 3 , 4

#### Materials examined.

***Holotype*.** Female. Indonesia, East Kalimantan Prov., Berau Ditrict, 1 km off the Tanjungredeb–Tnajungselor road, ca 45 km N of Tanjungredebm 2°29.5'N, 117°28.766'E, 190 m elev., primary forest, 29 September 2008, P. Schwendinger leg. ***Paratypes*.** One female and eight males, with the same collection data as the holotype.

#### Diagnosis.

Dorsal shield bearing smooth setae except two pairs of apically pilose setae near caudal margin. Surface of dorsal shield smooth, but web-like sculptural pattern situated anterior and posterior to eye-like dorsal depressions. Male sternal shield anterior to genital opening, and female genital shield covered by web-like sculptural pattern.

#### Description.

**Female** (n = 2). Length of idiosoma 1570–1610, width at level of coxae IV 1130–1145, colour reddish-brown. Shape of idiosoma pentagonal, its caudal margin curved.

**Figure 1.**
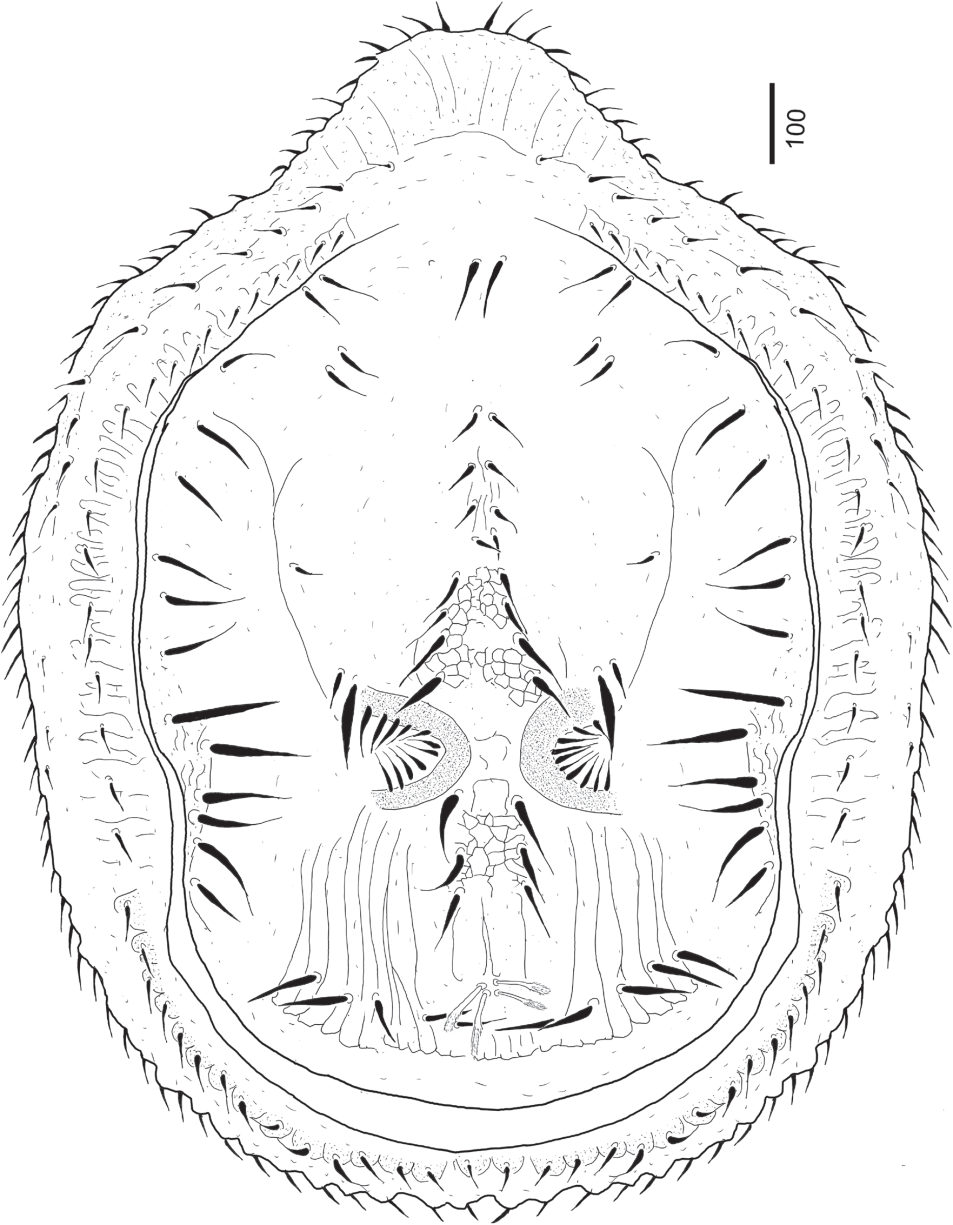
Dorsal view of *Bardizoneotvosi* sp. nov., holotype, female.

***Dorsal idiosoma*** (Figs 1, 4A, B). Marginal and dorsal shields fused anteriorly. Central area elevated from neighbouring regions on dorsal shield (Fig. 4B). One pair of eye-like depressions on elevated central part; margins of depressions covered by smooth, short (ca 42–45), needle-like setae. Majority of dorsal shield with smooth surface; web-like sculptural pattern situated only anterior and posterior to eye-like dorsal depressions on central area and some longitudinal lines present posterior to eye-like depressions. Dorsal shield bearing 35–38 pairs of smooth (ca 70–124 long) and two pairs of apically pilose (ca 75–80 long) setae. Longer (ca 110–125), smooth setae at level of oval depressions and near lateral margin of dorsal shield; apically pilose setae near posterior margin of dorsal shield. Marginal shield with some rounded platelets bearing short (ca 18–22), smooth setae on laterocaudal area and some reticulated sculptural pattern on marginal shield anterior to platelets. Other setae on marginal shield similar in shape and length to setae situated on platelets.

**Figure 2.**
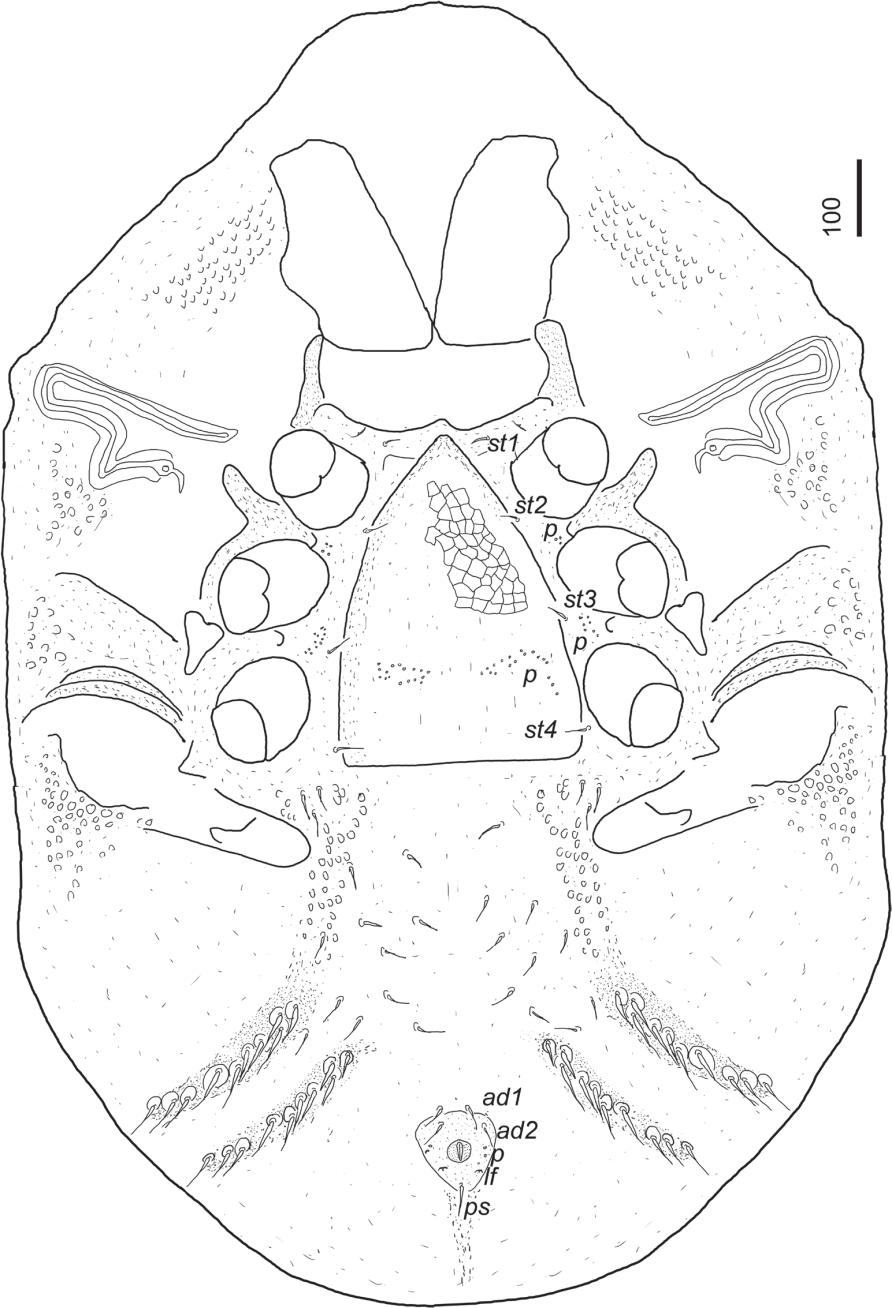
Ventral view of *Bardizoneotvosi* sp. nov., holotype, female.

***Ventral idiosoma*** (Figs 2, 4C, D). Four pairs of sternal setae present. All sternal setae smooth, needle-like, and ca 23–28 long. Setae *st1* inserted near anterior margin of sternal shield; *st2* at level of posterior margin of coxae II; *st3* at level of posterior margin of coxae III; *st4* at level of posterior margin of coxae IV. Sternal shield smooth, two pairs of field of poroid-like structures between coxae II and III and coxae III and IV. One pair of lyriform fissures visible near *st1*. About 13–16 pairs of ventral setae short (ca 25–34), smooth, and needle-like. About 10–14 pairs of smooth, ca 41–48 long and needle-like setae placed on small platelets.; these setae on two pairs of strongly sclerotized, slightly elevated ventral grooves posterior to pedofossae IV. Surface of ventral shield ornamented by oval pits posterior to coxae IV; other part of surface smooth. Anal opening oval (30–32 long and 28–30 wide); anal valves smooth, without euanal setae. Adanal (ca 27–29 long) and postanal (ca 38–41 long) setae smooth and needle-like. Two pairs of poroid-like structures and one pair of lyriform fissures situated lateral to anal opening. Anal area slightly elevated from neighbouring regions.

**Figure 3.**
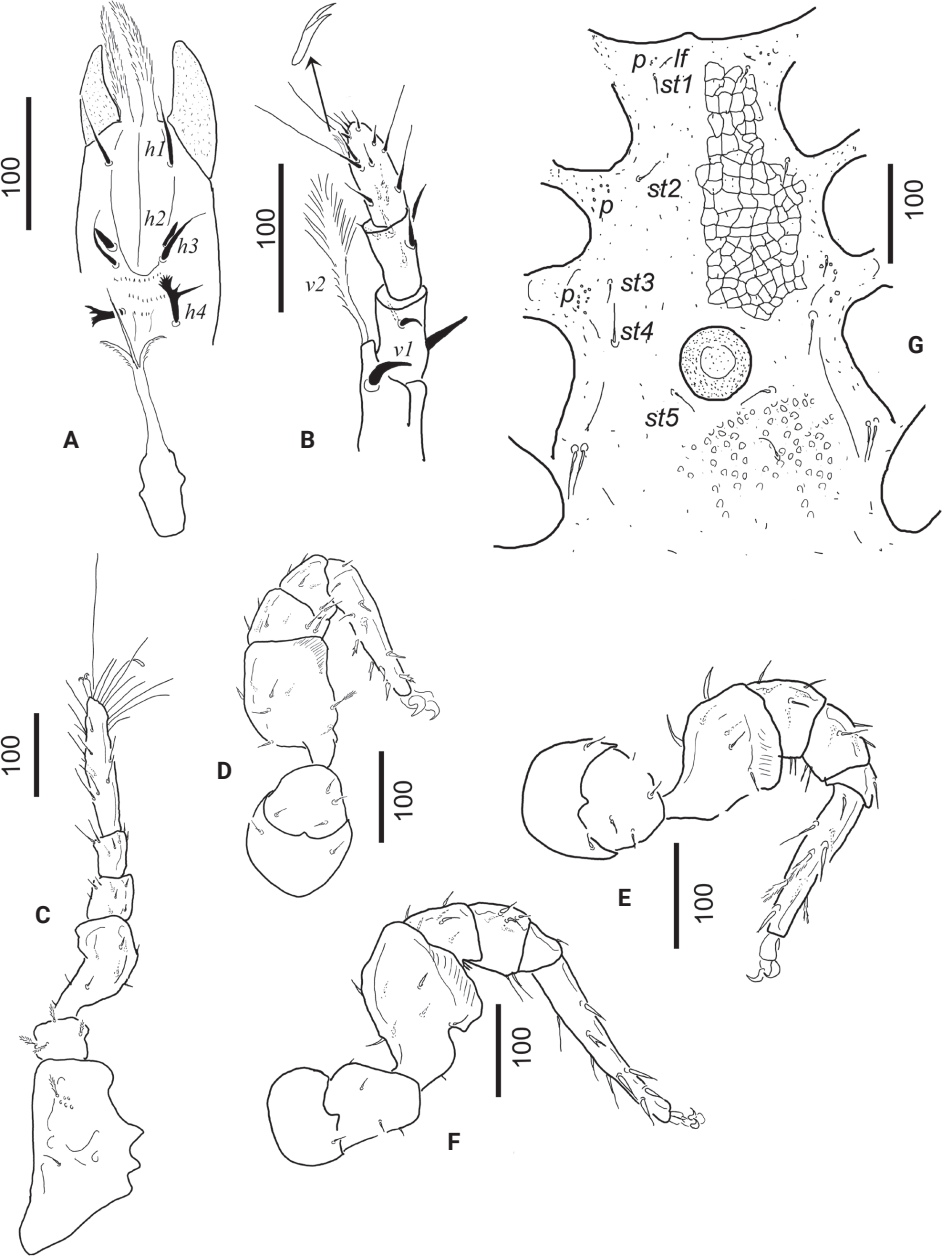
Dorsal view of *Bardizoneotvosi* sp. nov., holotype, female **A** ventral view of gnathosoma **B** ventral view of palp (arrow shows the palp apothele) **C** leg I in ventral view **D** leg II in ventrolateral view **E** leg III in ventrolateral view **F** leg IV in ventrolateral view **G** intercoxal area of male paratype.

Genital shield scutiform, length 430–440, basal width 300–315, situated between coxae II and IV; surface of genital shield covered by web-like structures. Stigmata situated between coxae II and III. Presitgmatid part of peritremes with two bends; postsigmatid part very short. Pedofossae deep, their surface smooth, with separate furrow for tarsi IV. Some oval pits situated outside margin of pedofossae. Tritosternum with narrow base; its laciniae subdivided into two pilose lateral branch and one smooth central branch (Fig. 3A).

***Gnathosoma*** (Fig. 3A, B). Corniculi smooth and horn-like; internal malae narrow and pilose, longer than corniculi. Hypostomal setae *h1* and *h3* smooth and needle-like (48–55 long); *h2* short (ca 16–18) and robust; and *h4* (36–39) antler-shaped. Deutosternal groove wide until *h3*, afterwards narrow; three rows of denticles posterior to setae *h3*. Chelicerae with internal sclerotized nodes. One central teeth situated on both cheliceral digit,, fixed digit as long as movable digit. Palp trochanter setae *v1* short and robust (ca 32–34); *v2* very long and pilose (ca 118–122). Other setae on palp segments smooth. Palp apothele with two branches (Fig. 3B). Epistome marginally pilose.

***Legs*** (Fig. 3C–F). Length of legs (from base of coxae to apex of tarsi): I 665–680, II 525–540, III 485–500, IV 505–518. Leg I with ambulacral claws, but shorter than other legs. On all legs majority of setae needle-like, but some setae serrate and several setae pilose on other leg segments.

**Male** (n = 8). Body 1570–1610 long and 1090–1115 wide at level of coxae.

***Dorsal idiosoma*.** As for the female.

**Figure 4.**
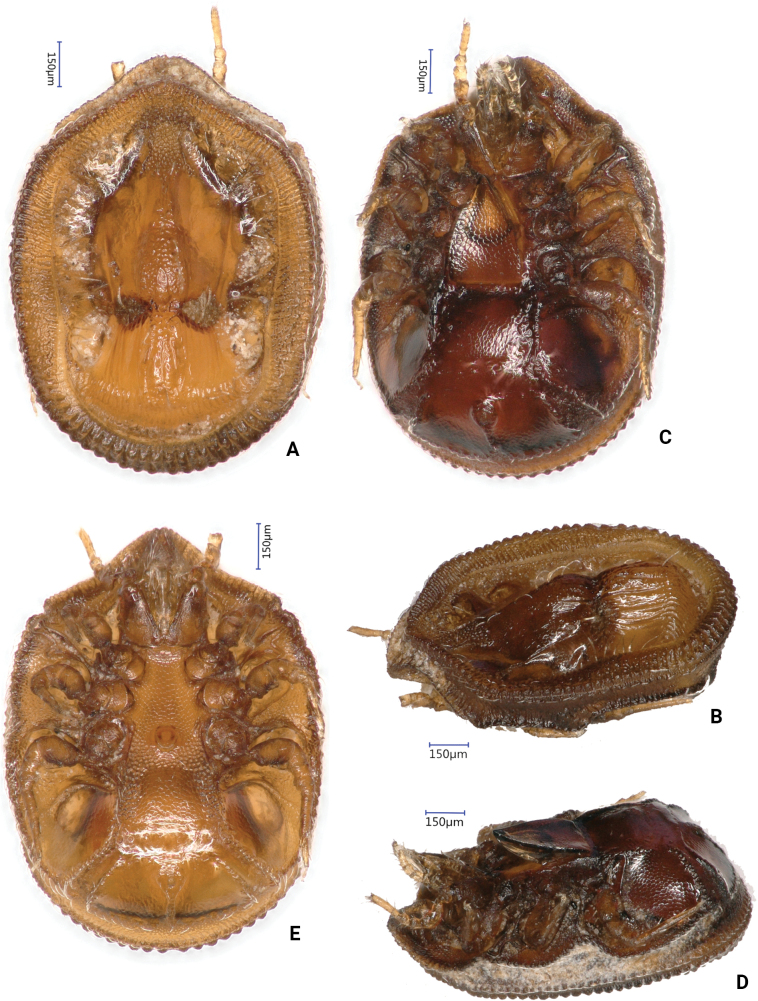
Photos about *Bardizoneotvosi* sp. nov., holotype, female **A** dorsal view of idiosoma **B** dorsolateral view of idiosoma **C** ventral view of idiosoma **D** lateroventral view of idiosoma **E** ventral view of idiosoma of male paratype.

***Ventral idiosoma*** (Figs 3G, 4E). Intercoxal area, with sternal setae and genital shield as in Fig. 3G. Sternal setae smooth and needle-like. Setae *st1* (ca 16–18) near anterior margin of sternal shield; *st2* (ca 25–26) at level of posterior margin of coxae II; *st3* (ca 26–28) at level of posterior margin of coxae III; *st4* (ca 30–84) at level of central area of coxae IV; *st5* (ca 25–27) near posterior margin of genital shield. Surface of sternal shield with web-like sculptural pattern anterior to genital opening and with oval pits posterior to genital opening. One pair of lyriform fissures and one pair of poroid-like structures near *st1*, two pairs of field of poroid-like structures between coxae II and III and coxae III and IV. Genital shield rounded (ca 70–73 × 67–69), its surface smooth, without eugenital setae, and situated between coxae IV.

Other characters as in female.

**Developmental stages.** Unknown.

#### Etymology.

The new species is dedicated to Baron Loránd Eötvös (1848–1919), scientist, physicist, the president of the Hungarian Academy of Sciences (1889–1905) and Minister of the Culture (1894–1895) on the 125^th^ anniversary of his birth.

#### Remarks.

The new species is most similar to *B.akoii* (Hiramatsu, 1985), the most important differences being summarized in Table 1.

**Table 1.** Most important differences between the species *Bardizonaokii* and *B.eotvosi* sp. nov.

	*B.aokii*	*B.eotvosi*
Majority of dorsal setae	finely pilose	smooth
Surface of anterior area of dorsal shield	with oval pits	smooth
Surface dorsal shield anterior and posterior to the eye-like depressions	smooth	with web-like sculptural pattern
Oval pits posterior to coxae IV	absent	present
Surface of male sternal shield anterior to genital opening	smooth	with web-like sculptural pattern
Surface of male sternal shield posterior to genital opening	smooth	with oval pits
Apical bend of peritreme	wide and angular	hooked

### ﻿Key to the known species of the family Phymatodiscidae

**Table:** 

1	Dorsal idiosoma with one pair of eye-like depression (genus *Bardizon*)	**2**
–	Dorsal idiosoma without eye-like depressions (genus *Phymatodiscus*)	**8**
2	Surface of female genital shield smooth, only bearing some pits	**3**
–	Surface of female genital shield ornamented with web-like sculptural pattern	**7**
3	Eye-like transversal furrows large, visible, and bordered with long setae	**4**
–	Eye-like transversal furrows small, hidden, and not bordered with setae	***B.insolitus***
4	Dorsal setae uniform in length	**5**
–	Dorsal setae not uniform in length	**6**
5	Setae on marginal shield situated in multiple rows; two pairs of long and narrow setae on caudal area of dorsal shield	***B.haradai***
–	Setae on marginal shield situated in only one row; two pairs of robust setae on caudal area of dorsal shield	***B.oculatus***
6	Setae *h1* marginally serrate; setae on margin of eye-like transversal furrows smooth	***B.kuni***
–	Setae *h1* smooth; setae on margin of eye-like transversal furrows marginally pilose	***B.malayicus***
7	Surface of anterior area of dorsal shield without oval pits	***B.eotvosi***
–	Surface of anterior area of dorsal shield with oval pits	***B.aokii***
8	Margin of idiosoma with several long prolongations	**9**
–	Margin of idiosoma without prolongation	***P.polyglottis***
9	Marginal prolongations situated only on caudal margin	***P.iriomotensis***
–	Marginal prolongations situated on entire margin	**10**
10	Prolongations cone-like	**11**
–	Prolongations not cone-like	**12**
11	Margin with more than 14 prolongations	***P.mirabilis***
–	With fewer than 14 prolongations	***P.coniferus***
12	Margin with more than 14 prolongations	***P.mirandus***
–	With fewer than 14 prolongations	***P.ignesemovens***

## Supplementary Material

XML Treatment for Phymatodiscidae

XML Treatment for Phymatodiscus

XML Treatment for Bardizon

XML Treatment for Bardizon eotvosi

## ﻿Additional information

### Author ORCIDs

Jenő Kontschán https://orcid.org/0000-0001-8274-4238

Sergey G. Ermilov https://orcid.org/0000-0002-0913-131X

### Data availability

All of the data that support the findings of this study are available in the main text.
